# Added value of 3D-DRIVE and SWI Magnetic Resonance Imaging Sequences in Intraventricular Neurocysticercosis (IVNCC): An Institutional Experience from Northeast India

**DOI:** 10.15388/Amed.2021.28.2.21

**Published:** 2021-12-29

**Authors:** Deb K. Boruah, Bidyut Bikash Gogoi, Kuntal Kanti Das, Kalyan Sarma, Pranjal Phukan, Binoy Kumar Singh, Karuna Hazarika, Awadhesh Jaiswal

**Affiliations:** Department of Radio-diagnosis, Lakhimpur Medical College, Assam, India; Department of Pathology, Assam Medical College, Dibrugarh, Assam, India; Department of Neurosurgery, Sanjay Gandhi Postgraduate Institute of Medical Sciences, Lucknow, Uttar Pradesh, India; Department of Neuroradiology, AIIMS, New Delhi, India; Department of Radiodiagnosis, North Eastern Indira Gandhi Regional Institute of Health & Medical Sciences, India; Department of Neurosurgery, North Eastern Indira Gandhi Regional Institute of Health & Medical Sciences, India; Department of Radio-diagnosis, Tezpur Medical College, Assam, India; Department of Neurosurgery, Sanjay Gandhi Postgraduate Institute of Medical Sciences, Lucknow, Uttar Pradesh, India

**Keywords:** 3DT2W-driven equilibrium radiofrequency reset pulse (DRIVE), magnetic resonance imaging, susceptibility weighted imaging (SWI), intraventricular neurocysticercosis (IVNCC)

## Abstract

**Background:**

Prompt diagnosis and early treatment institution are important in intraventricular neurocysticercosis (IVNCC) as compared to the parenchymal or racemose form because it is associated with a poorer patient prognosis. Intraventricular neurocysticercosis is often missed on CT scan or conventional cranial magnetic resonance imaging because of similar density or signal intensity of cysticercus lesion with cerebrospinal fluid.Thestudy aims to evaluate the added value of 3D-DRIVE and SWI MRI sequences in isolated intraventricular cysticercosis with acute neurological presentation.

**Methods and Materials:**

This retrospective study was carried out on diagnosed 10 patients with isolated intraventricular neurocysticercosis (IVNCC) presented to a tertiary care hospital with an acute onset of symptoms or acute neurological deficit between June 2019 to May 2021. Relevant neurological examination, CSF analysis, a serological test of neurocysticercosis and MRI scan of the brain were performed.

**Result:**

Tenpatients of isolated intraventricular neurocysticercosis (3 males and 7 females) having 3 pediatric and 7 adults were included in this study sample.The common neurological complications of the isolated intraventricular neurocysticercosis in this study are observed as obstructive hydrocephalus in 8(80%) patients and ependymitis in 7(70%) patients. IVNCC with distinctly visualized scolex (visibility score 2) identified in 2(20%) patients in T2WI, 8 (80%) patients in 3D-DRIVE and 3(30%) patients in SWI sequences. The cyst wall of IVNCC was distinctly visualized (visibility score 2) in 1(10%) patient in T2WI, 8(80%) patients in 3D-DRIVE and 6(60%) patients in SWI sequence.

**Conclusion:**

Heavily T2-weighted steady-state and SWI sequences should be added to routine MRI sequences that helps to identify IVNCC and should be used in patients with unexplained hydrocephalus, especially in endemic regions of Neurocysticercosis**.**

## Abstract (lt)

**Papildoma 3D DRIVE ir SWI (svertinio jautrumo vaizdavimo) magnetinio rezonanso tomografijos sekų nauda intraventrikulinės neurocisticerkozės (IVNCC) atveju. Šiaurės Rytų Indijos įstaigų patirtis:**

**Santrauka. *****Problema. ***Intraventrikulinės neurocisticerkozės (IVNCC) atveju, kitaip nei sergant parenchimine ar subarachnoidine forma, svarbu ligą greitai diagnozuoti ir taikyti ankstyvą gydymą, nes nuo to priklauso pasveikimo prognozė. Intraventrikulinė neurocisticerkozė dažnai nenustatoma atliekant KT tyrimą ar įprastą kaukolės magnetinio rezonanso tomografiją, nes cisticerkinio pažeidimo bei galvos ir stuburo smegenų skysčio tankis ir signalo intensyvumas yra panašūs. Tyrimo tikslas – įvertinti papildomą 3D DRIVE ir SWI MRT sekų naudą ūminės neurologinės izoliuotos intraventrikulinės cisticerkozės atveju.

***Metodai ir medžiaga. ***Atliktas retrospektyvus 10 pacientų, kuriems diagnozuota izoliuota intraventrikulinė neurocisticerkozė (IVNCC), atvykusių į trečio lygio ligoninę 2019 m. birželio mėn. – 2021 m. gegužės mėn. pasireiškus ūminiams simptomams ar ūminiam neurologiniam deficitui. Atlikta atitinkama neurologinė apžiūra, galvos ir stuburo smegenų skysčio tyrimas, serologinis neurocisticerkozės tyrimas ir galvos smegenų MRT.

***Rezultatas. ***Tyrime dalyvavo dešimt pacientų, sergančių izoliuota intraventrikuline neurocisticerkoze (3 vyrai ir 7 moterys, 3 vaikai ir 7 suaugusieji). Šio tyrimo metu nustatytos izoliuotai intraventrikulinei neurocisticerkozei būdingos neurologinės komplikacijos – obstrukcinė hidrocefalija 8(80 %) pacientams ir ependimos uždegimas 7(70 %) pacientams. IVNCC su aiškiai matomu skoleksu (2 matomumo balas) nustatytas 2 (20 %) pacientams taikant T2WI, 8 (80 %) pacientams taikant 3D DRIVE ir 3 (30 %) pacientams taikant SWI sekas. IVNCC cistos sienelės aiškiai matomos (2 matomumo balas) 1 (10 %) pacientui taikant T2WI, 8 (80 %) pacientams taikant 3DDRIVE ir 6 (60 %) pacientams taikant SWI sekas.

***Išvada.*** T2WI ir SWI sekos turėtų būti atliekamos su įprastomis MRT sekomis, siekiant nustatyti IVNCC, ir turėtų būti atliekama pacientams, kuriems dėl neaiškių priežasčių pasireiškia hidrocefalija, ypač tuose regionuose, kuriuose siaučia neurocisticerkozės endemija.

**Raktažodžiai: **3DT2W (DRIVE), magnetinio rezonanso tomografija, svertinis jautrumas (SWI), intraventrikulinė neurocisticerkozė (IVNCC)

## Introduction

The incidence of intraventricular neurocysticercosis (IVNCC)was reported as 7.3% to 61.3% in neurocysticercosis [1, 2]. The fourth ventricle is the most common site for intraventricular neurocysticercosis (43–70%), followed by lateral ventricle (11–43%), third ventricle (1–29%) and aqueduct of Sylvius (7–9%) [1, 3, 4, 5, 6]. About 38% of patients of IVNCC presented with rapid neurological detoriation and acute neurological deficit and even sudden death [7].

In intraventricular neurocysticercosis, the larvae of Taeniasolium reach the cerebral ventricles via the choroid plexus and causes symptoms from CSF flow obstruction toependymitis, ventriculitis, or both [1, 2]. 

Most of the IVNCC is usually occult or missed on CT scan owing to attenuation characteristics of cysticercus cyst fluid equal to those of CSF. Neurocysticercus lesions in the CSF spaces are usually missed on conventional MRI sequences. Sometime post-gadolinium MRI images are not able to characterize the IVNCC because the signal intensity of the cyst is similar to that of CSF [8]. 

It has been shown that heavily T2-weighted high-resolution steady-state sequences like 3DDRIVE/3D-CISS/FIESTA better delineates the cysticercus lesions in cisternal spaces, cerebral sulci or within the ventricle [9]. The 3D-DRIVE sequence is a 3D T2-weighted driven equilibrium radio-frequency reset pulse. CISS is a heavily T2W constructive interference in steady-state sequenceand FIESTA is a fast imaging employing steady-state acquisition.

These heavily T2-weighted images increase the conspicuity of the lesion within a CSF space. So it is able to identify the cyst wall or scolex, which are not otherwise seen on conventional MRI [10].

Susceptibility weighted imaging (SWI) sequence is a 3D gradient-echo sequence with both magnitude and phase information, it separately and in combination provides additional information in identifying the calcified scolex of intraventricular cysticercus lesion in comparison to the conventional MRI sequences [11, 12].

Advanced MRI techniques can be able to identify the adherence of IVNCC into the wall of the ventricle or able to detect ventriculitis, ependymitis and obstructive hydrocephalus. Identification of adherent IVNCC is of utmost importancein MRI imaging, as even surgical resection sometimes fails to treat it, and in such situation it may need a CSF diversion with or without cyst excision [1, 13].

Intraventricular Neurocysticercosis (IVNCC) had a poorer prognosis than other forms of Neurocysticercosis [14]. The active viable stage of Intraventricular Neurocysticercosis doesn’t produce a reaction to the host, however, it may produce noncommunicating hydrocephalus.With the death of intraventricular larva, the host reaction is induced in the form of ependymitis, ventriculitis and meningoencephalitis. 

The common alarming symptoms of IVNCC include acute onset of headache, vomiting, decreased visual acuity, altered mental status and even death [1, 4, 5]. Isolated intraventricular neurocysticercosis, especially in the fourth ventricle, can cause mechanical obstruction of CSF flow subsequently resulting in hydrocephalus [4, 5]. The inflammatory process in IVNCC varies according to the location of the parasite [15]. MRI with newer advanced techniques is necessary to locate the IVNCC prior to surgery, as sometime IVNCC are mobile and migrated within the ventricular system. 

The study aims to evaluate the added value of 3D-DRIVE and SWI MRI sequences in acute neurological complications of isolated intraventricular cysticercosis.

## Methods and Materials

***Patient selection*****: **The case records of the 10 patients were retrospectively analyzed from June 2019 to May 2021. All patients of isolated intraventricular neurocysticercosis (IVNCC) presented with features of raised intracranial pressure with the onset of symptoms of headache, vomiting or acute neurological deficit. This retrospective study was approved by the institutional ethics review committee.

***Inclusion criteria*** **:**

1. Only isolated intraventricular neurocysticercosis with acute clinical presentation.

***Exclusion criteria:***

1. Intraventricular neurocysticercosis along with a parenchymal or racemose form of neurocysticercosis.

***MRI Protocol: ***All 10 patients underwent an MRI scan of the brain, using a 1.5 T MR scanner**, ** Philips Ingenia (Philips Medical System, The Netherlands). MRI scans of the brain was done using a dedicated 32 channel head coil**. **Conventional MRI sequences include axial T1WI, T2WI, FLAIR (fluid-attenuated inversion recovery), DWI (diffusion-weighted imaging), sagittal T1WI and coronal T2WI. 3D-DRIVE (3D T2-weighted driven equilibrium radiofrequency reset pulse) and SWI (susceptibility-weighted imaging) sequences were obtained, followed by post-gadolinium T1WI sequences in all three planes. The parameters of the various MRI sequences used are shown in Table 1. 

**Table 1.** showing parameters used in various MRI sequences

MRI sequence	TE(ms)	TR(ms)	Matrix	Field of view (FOV)	Slice thickness (mm)	Flip angle	Others
**T2W axial**	90–110	3500–4500	512	220–250	5	90°	
**T1W axial**	10–15	450–650	512	220–250	5	90°	
**FLAIR axial**	100–140	9000–11000	512	220–250	5	90°	TI=2500–2800ms
**DWI axial**	9–10	3000–4000	160 × 100	220–250	5	90°	b-value = 1000s/mm^2^
**SWI axial**	10–24	50–60	512	220–250	2	20°	
**T1W-sagittal**	10–15	450–650	512	220–250	4	90°	
**T2W-coronal**	90–110	3500–4500	512	220–250	4	90°	
**3D-DRIVE**	8–10	1500	512	140–160	1.1	90°	
**Post-contrastT1W_SPIR axial, coronal & sagittal**	10–15	450–650	512	220–250	4	90°	I.V. Gadolinium 1ml/kg bodyweight

***MRIEvaluation*****: **MRI images were evaluated for the location and size of the intraventricular neurocysticercosis. The presence of a scolex was looked for in the T1WI, T2WI, 3D-DRIVE and SWI images. Presence of cyst wall or wall calcification was also looked for in SWI and 3D-DRIVE sequences. The maximum wall thickness of the intraventricular cyst was measured in the 3D-DRIVE sequence. The pattern of post-contrast enhancement of the IVNCC, associated ependymitis, meningitis, hydrocephalus and entrapment of ventricle were also observed. 

***Visibility score of Intraventricular neurocysticercosis (IVNCC)*****:** The visibility of the wall and scolex of the intraventricular neurocysticercosis was categorized on a 3-point scale from 0 to 2. Score 0 means “notdetected”, 1 means “probablyseen” and 2 means “distinctlyseen”. To know the diagnostic performance of T2W, 3D-DRIVE and SWI sequences in the detection of intraventricular neurocysticercosis, this visibility score was calculated. 

***Neurological assessment of intraventricular neurocysticercosis(IVNCC)*****:** The details of clinical and neurological examinations performed in all patients. 

***Follow up and final diagnosis*****:** Craniotomy with microsurgical cyst excision was done in 2 patients and neuroendoscopic cyst excision in another 4 patients, and histopathological confirmation of cysticercus lesions were confirmed. Three patients were confirmed having IVNCC on subsequent follow up MRI scans on basis of regression in size or complete healing of cysticercus lesion, with initial positive IgM antibodies against cysticercus antigens measured by ELISA technique both in CSF and Serum serology. Another 1 patient was diagnosed with IVNCC only basis of initial MRI imaging findings of intraventricular cyst with positive IgM antibodies against cysticercus antigens both in CSF and Serum.

All 10 patients received anticysticercal therapy with albendazole. Patients were followed up for a period of 6 months to 18 months.

**Statistical analysis**: All statistical analysis was performed using Statistical Package for Social Science (SPSS, version 16). Data were presented in terms of percentage and mean. A Chi-square test is done to find out the sensitivity and specificity of T2W, 3D-DRIVE and SWI sequences.

## Results

**Figure 1.**
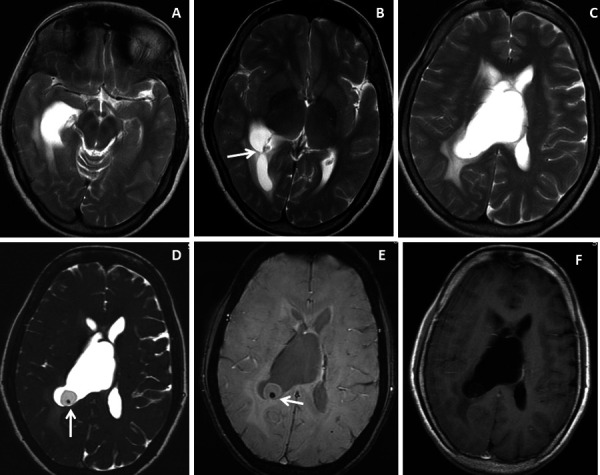
Brain magnetic resonance imaging of 52 years female with headache and vomiting. (A-C) Axial T2WI images show asymmetrical hydrocephalus with entrapment of temporal horn of the right lateral ventricle with narrowing / adhesion in the trigone of the lateral ventricle (arrow) with surrounding periventricular CSF seepage. (D) Heavily T2-weighted image shows an intraventricular neurocysticercus lesion with a T2 hypointense scolex within (arrow). (E) Axial susceptibility-weighted image shows nodular blooming of the calcified scolex within the thin wall IVNCC (arrow). (F) Axial post-gadolinium T1W image shows thin smooth peripheral cyst wall enhancement with the smooth ependymal enhancement of the asymmetrically dilated lateral ventricle.

**Figure 2.**
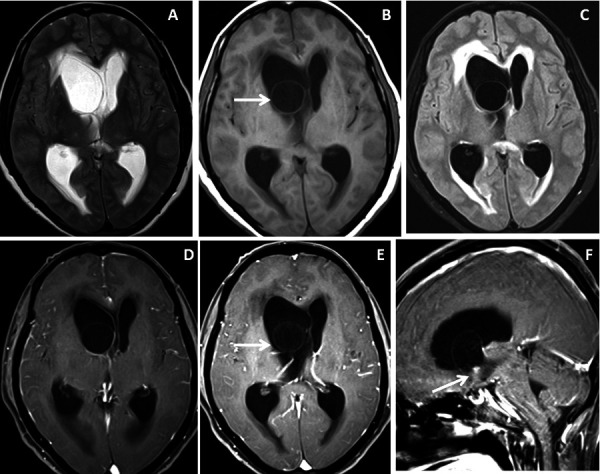
Brain magnetic resonance imaging of 50 years female with acute severe headache with acute neurological status and semi-comatose state. (A-C) Axial T2WI, FLAIR and T1WI images show a thin-walled T2 hyperintense cystic lesion in the frontal horn of the right lateral ventricle occluding the right-sided foramen of Monroe(arrow) with marked supratentorial hydrocephalus with periventricular CSF seepage. (D-F) Axial and sagittal post-gadolinium T1W images show smooth peripheral cyst wall enhancement (arrow) with a small enhancing mural nodule inferiorly (arrow in image F).

**Figure 3.**
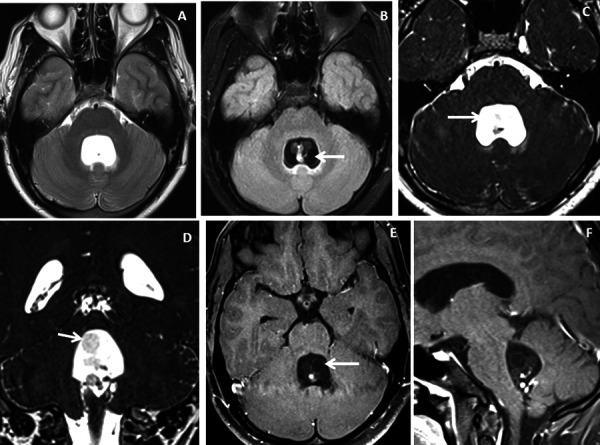
Brain magnetic resonance imaging of 25 years male with headache. (A-B) Axial T2WI and FLAIR images show distension and dilatation of the fourth ventricle with minimal periventricular CSF seepage. Variable signal intensity cystic lesions are seen within the fourth ventricle on the FLAIR image (arrow). (C-D) Axial and coronal heavily T2W images show multiple thin-walled hyperintense cystic lesions (arrows) with less hyperintense CSF signal within the 4^th^ ventricle with variable sizes irregular nodules within. (E-F) Axial and sagittal T1W post-contrast image shows thin smooth enhancement of the intraventricular cyst (arrow) with enhancing irregular nodules. Post-contrast enhancement is also seen along the ependymal lining of the 4^th^ ventricle, in posterior and inferior aspects.

**Figure 4.**
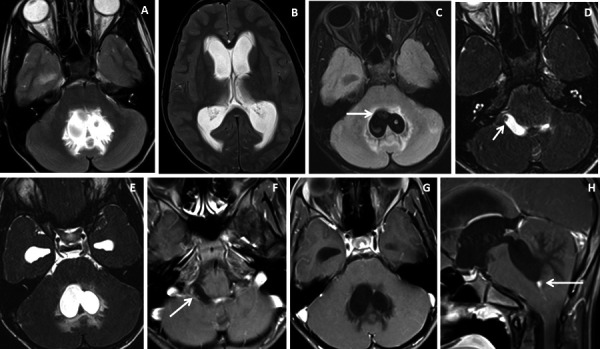
Brain magnetic resonance imaging of 13 years male with severe headache, vomiting and acute neurological deterioration. (A-B) Axial T2WI images show marked distension and dilatation of the fourth ventricle with marked periventricular CSF seepage and supratentorial hydrocephalus. (C) Axial FLAIR image shows cystic lesions within the fourth ventricle (arrow). (D-E) Axial heavily T2W images show thin-walled hyperintense cystic lesions within the 4^th^ ventricle with an oval to elongated appearing cystic lesion with scolex in the right foramen of Luschka (arrow). (F-H) Axial and sagittal T1W post-contrast image shows a thin peripheral enhancing cystic lesion with an irregular mural nodule in the right foramen of Luschka (arrow) and thin smooth enhancement of the intraventricular cysts. Nodular enhancement was seen in midline inferior margin of 4^th^ ventricle near to foramen of Magendie (arrow).

**Figure 5.**
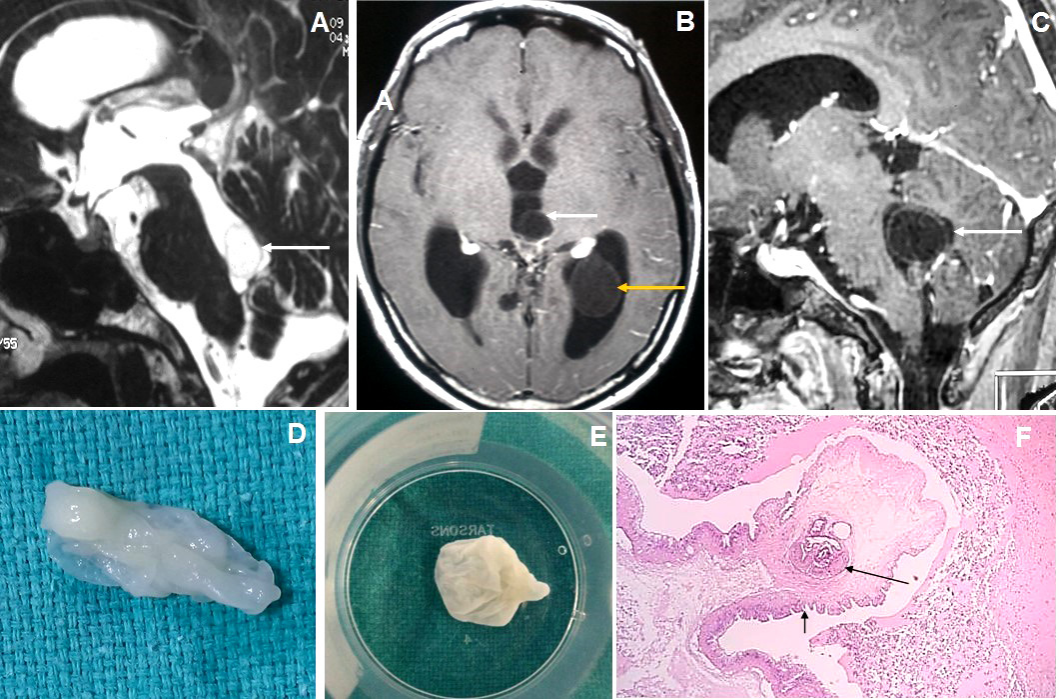
Brain magnetic resonance imaging of 80 years male with headache and visual disturbance. (A) Sagittal T2WI image shows a thin-walled T2 hyperintense cysticercus lesion in the fourth ventricle (arrow) causing supratentorial hydrocephalus. (B-C) Axial and sagittal T1W post-contrast images show cysticercus lesions within the 3rd ventricle (white arrow in image B), trigone of the left lateral ventricle (yellow arrow) and fourth ventricle (arrow in image C). (D-E) Neuroendoscopic post-excision cysticercus specimen shows thin-walled cystic lesions. (F) HPE image shows sucker (long arrow) and cuticle (short arrow) of the cysticercus lesion.

The study sample comprised of 10 patients (n=3 males and n=7 females) with a mean age of 37.2 ± 22.7 [SD] years and a male: female ratio of 1:2.3. Five (50%) patients presented with acute onset of headache and vomiting, 3(30%) with altered consciousness and neurological deterioration, 1(10%) patient had a seizure and another 1(10%) patient had visual disturbance. Intraventricular neurocysticercosis lesions were identified within the lateral ventricle in 5(50%) patients (Figure 1 and 2), fourth ventricle in 2(20%) patients (Figure 3 and 4) and third ventricle in 1(10%) patient (Figure 5). Two (20%) patients had intraventricular neurocyticercus lesions in more than one ventricle. Five (50%) patients had only solitary intraventricular neurocysticercus lesions, 1(10%) patient had two neurocysticercus lesions, 2 (20%) patients had three neurocysticercus lesions (Figure 5) and another 2(20%) patients had more than three intraventricular neurocysticercus lesions (Figure 3).

The mean diameter of the largest intraventricular cysticercosis was 18.2±6.9 mm [SD] and the mean wall thickness of intraventricular neurocysticercosis was 1.3±0.23 mm [SD]. 

Very thin smooth cyst wall enhancement was observed in 9(90%) patients (Figure 1 and 2) and irregular peripheral rim-like wall enhancement with an irregular nodule in 1(10%) patient. Ventricular ependymitis with smooth to irregular ependymal lining enhancement was observed in 7(70%) patients (Figure 3). Presence of hydrocephalus detected in 8(80%), where MRI revealed asymmetrical enlargement of lateral ventricle with periventricular CSF seepage in 4(40%) patients (Figure 1), dilatation of 3^rd^ ventricle in 2(20%) patients and ballooned 4^th^ ventricle in another 2(20%) patients (Figure 4). 

***T2WI, DRIVE and SWI visibility score of the scolex of intraventricular neurocysticercosis (IVNCC): ***In 10patients with intraventricular neurocysticercosis, the scolex was distinctly visualized (visibility score 2) in 2(20%) patients on T2WI, 8(80%)patientson 3D-DRIVE (Figure 1) and 3(30%) on SWI sequence (Figure 1). For identification of scolex in intraventricular neurocysticercosis with a visibility score of 2, the 3D-DRIVE sequence had a sensitivity of 80% followed by 30% with SWI and 20% with T2W images. The poorly visualized scolex of intraventricular neurocysticercosis (visibility score 1) noted in 2 (20%) patients on 3D-DRIVE, 5(50%)patientson SWI and 2(20%) on T2W sequence [Table 2]. 

**Table 2.** Visibility score of cyst wall and scolex of intraventricular neurocysticercosis on T2WI, 3D-DRIVE and SWI sequences in 10 patients.

MRI sequence	Visibility score	Cyst wall	Scolex	p-value (chi-square)
**Axial T2WI**	Score 2	1	2	0.323
	Score 1	4	2	
	Score 0	5	6	
**3D-DRIVE**	Score 2	8	8	0.002
	Score 1	2	2	
	Score 0	0	0	
**SWI**	Score 2	6	3	0.329
	Score 1	4	5	
	Score 0	0	2	

***T2WI, DRIVE and SWI visibility score of the wall of intraventricular neurocysticercosis: ***Of 10patients with IVNCC, the cyst wall was distinctly visualized (visibility score 2) in 1 (10%) patient on T2WI, 8 (80%) patientson 3D-DRIVE (Figure 1 and 2) and 6 (60%) patients on SWI sequence. For identification of wall of IVNCC with a visibility score of 2, the 3D-DRIVE sequence had a sensitivity of 80% followed by 60% with SWI and 10% with T2W images. Cyst wall calcification was identified in 1(10%) patient on SWI. Poorly visualized wall of intraventricular neurocysticercosis (visibility score 1) noted in 2(20%) patients on 3D-DRIVE, 4(40%) patients on SWI and 4(40%) on T2W sequences [Table 2].

Four (40%) patients were treated conservatively with cysticidal drugs only and 6 (60%) patients were treated surgically followed by cysticidal drugs. Craniotomy with micro-surgical resection was done in 2 patients with 4^th^ ventricular neurocysticercosis and endoscopic approach in another 4 patients with lateral and 3^rd^ ventricular neurocysticercosis. On follow-up, 8(80%) patients show complete healing of the intraventricular neurocysticercosis, 1 (10%) patient showed disease recurrence and another 1 (10%) patient died. 

## Discussion

Acute onset of headache, vomiting, altered mental status and visual disturbance can occur in intraventricular neurocysticercosis (IVNCC) [16]. Acute onset of hydrocephalus can lead to sudden death due to brain stem herniation, displacement or distortion[17]. This sudden onset of symptoms is associated with changes in patient’s head position, because acute ventricular obstruction can occur in Bruns’ syndrome [18, 19]. It occurs due to intermittent CSF flow obstruction by the ball-valve movement of intraventricular cysts. Intraventricular neurocysticercosis occurs in approximately 20% of patients of neurocysticercosis worldwide [20]. It carries high mortality rate due to acute onset of hydrocephalus and neurological detoriation. The viable intraventricular neurocysticercosis is often freely mobile and may lodge in ventricular foramina like foramen of Monroe, Luschka, Magendie or aqueduct of Sylvius, and lead to acute obstructive hydrocephalus [21]. Isolated fourth IVNCC is associated with ependymitis, arachnoiditis and ventriculitis [21]. 

CT scan fails to identify IVNCC that do not deform the ventricle because of same density of cyst with CSF, cyst wall and scolex not visible or cyst wall not shows any abnormal wall enhancement[22, 23, 20, 21]. MRI can able to identify IVNCC in approximately 80% of cases [24] with a classical intraventricular cyst with scolex [25]. 

MRI appearance of an intraventricular cystic lesion with T1-weighted hyperintense or T2-weighted hypointense scolex within are considered as pathognomonic for intraventricular neurocysticercosis and these characteristics are usually not detected by CT scan. Conventional MRI sequences routinely fails to identify the scolex or cyst wall. Hence additional newer advanced MRI sequences like 3D-DRIVE and SWI act as a solving tool in the identification and characterization of intraventricular cystic lesions [10]. 

The common differential diagnosis of intraventricular NCC included colloid cyst, ependymal cyst, choroidal plexus cyst, intraventricular epidermoid and arachnoid cyst. And to differentiate these intraventricular cystic lesions newer advanced MRI techniques are necessary [Table 3]. 

**Table 3.** Common differential diagnosis of isolated intraventricular neurocysticercosis (IVNCC) on MR imaging.

	IV NCC	Ependymal Cyst	Choroid plexus cyst	Intraventricular epidermoid cyst	Intraventricular arachnoid cyst	Colloid cyst	Intraventricular Cystic neoplasm
**Age**	-Any age -Commonest in 15-40 years	-Young adult -around 3^rd ^decade	-Common in neonates -Uncommon in adults	4-6^th^ decade	Any age	3-4^th^ decade	Adult -Cystic astrocytoma Children -cystic me-dulloblastoma
**Clinical** **presentations**	headache, vomiting, decreased visual acuity, altered mental status and even death	- asymptomatic - symptomatic in larger cyst	- Usually asymptomatic -Symptomatic in larger cysts	-asymptomatic -headache -cognitive deficit -larger produces mass effect, hydrocephalus	- usually asymptomatic -symptomatic with larger cyst - headache, hydrocephalus - hemorrhage can occur within a cyst	- usually asymptomatic -Headache -rarely present with acute obstructive hydrocephalus -sudden death[10]	- asymptomatic - Headache - acute obstructive hydrocephalus
**Location**	fourth ventricle followed by lateral, third ventricle and aqueduct of sylvius	- common in frontal horn of lateral ven-tricle[10]	Located in choroid plexus in posterior body of lateral ventricle. Usually bilateral[10]	4^th^ ventricle followed by 3^rd^ and lateral ventricle	- common in lateral ventricle followed by 3^rd^ and 4^th ^ventricle	anterior 3^rd ^ventricle followed by near to foramen of Monroe	4^th^ ventricle
**Conventional MRI**	-similar to CSF -Slightly higher signals on FLAIR compared to CSF.	Similar to CSF signals in all MRI sequences	Thin-walled cyst showing slightly to moderately hyperintense to CSF on FLAIR	Slightly higher signals on FLAIR compared to CSF.	Similar to CSF signals in all MRI sequences	Hypointense on T2W and hyperintense on T1W	- central T2 hyperintense with peripheral T2 iso to hypointense irregular wall or eccentric solid appearing component
**DWI**	No diffusion restriction	No diffusion restriction	2/3^rd^ showed diffusion restriction	Shows diffusion restriction with variable ADC value	No diffusion restriction	No diffusion restriction	Peripheral wall or solid component showed restriction
**Scolex**	Present	Absent	Absent	Absent	Absent	Absent	Absent
**Wall**	Smooth thin wall	Smooth wall	Smooth or irregular	Thin or may not seen	Thin or imperceptible	Not seen	Thick and irregular enhanced walls
**Hydrocephalus**	Common	Uncommon	- uncommon -larger lesion may cause hydrocephalus	- Uncommon - Rarely larger lesion causes hydrocephalus	Uncommon	- acute hydrocephalus is common	Common

The colloid cyst typically showed T1-weighted hyperintensities and was classically located in the anterior third ventricle or foramen of Monroe region. CT scan shows hyperdensity within the colloid cyst. Ependymal cyst is difficult to differentiate from an adherent intraventricular NCC, but ependymal cyst wouldn’t show scolex. The common location of ependymal cyst is in the frontal horn of lateral ventricle, where the cystis located near tothe foramen of Monroe region causing obstructive hydrocephalus. The choroid plexus cyst is usually asymptomatic and confines in the posterior body of the lateral ventricle. Intraventricular epidermoid cyst showed typical diffusion restriction without any scolex. Intraventricular arachnoid cyst suppressed signals on FLAIR images with an absence of scolex. 

IVNCC withoutabnormal post-contrast enhancement should be treated surgically with either neuroendoscopic or open surgery for cyst excision. Those IVNCC that showa peripheral rim-like post-contrast enhancement with or without adjacent ependymitis should probably undergo the CSF flow diversion with VP shunt or partial cyst excision with the CSF diversion. IVNCC showing rim-like post-contrast enhancement denotes ependymitis;if it is surgically resected, patient is likely to develop hydrocephalus after surgery. 

The surgical treatment options for intraventricular NCC depend on the clinical presentation, location and stage of IVNCC. Usual endoscopic or open microsurgical removal of intraventricular NCC should be considered if there is a CSF obstruction or mass effect, or fourth ventricular cyst. 

Various recent literatures show application of various newer MRI sequences for identification and localization of IVNCC [26, 27]. Conventional MRI sequences are lesssensitive for identificationof IVNCC, however the previous study of Singh et al. [26] identified scolex on FLAIR and T1W images in 3 out of 4 patients of IVNCC, and T2W fails to identify the same. Govindappa SS et al. [10] found more accuracy of 3D-CISS than of conventional MRI sequences for identification of IVNCC. However, Robbani I et al.[28] and Mont’Alverne Filho FE et al. [9] found more accuracy of 3DSPGR (spoiled gradient recalled echo) overthat of conventional MRI sequences for identification of IVNCC. Table 4 shows few review literature of intraventricular neurocysticercosis. 

**Table 4.** Few review literature of intraventricular NCC on magnetic resonance imaging (MRI).

S/N	Study/year	No of patient of IVNCC	Location of IVNCC	Mean/ Median age(yrs.)	CEMRI enhancement Pattern	Complication	Treatment
1.	**Cuetter AC et al. / 2002(1)**	18	2-isolated IVNCC With extra-ventricular NCC-16	Mean = 35	-	Hydrocephalus-18	Medical treatment -8 Surgical Treatment -10
2.	**Citow JS et al. / 2002(3)**	30	30-IVNCC LV-5(16.7%) 3V-5(16.7%) 4V-21(70%) With extra-ventricular NCC-	Mean= 36	17-no enhancement 14- peripheral wall enhancement of the IVNCC	29	Surgical Treatment -30
3.	**Kumar R et al. / 2008(29)**	11	11-IVNCC LV-4(36.4%) FOM-2(18.2%) Aqueduct-1(9%) 4V-4(36.4%) With extra-ventricular NCC-0	Mean=14.2 ±2.98	-	Hydrocephalus-7	Surgical Treatment -11
4.	**Mont’Alver-ne Filho FE et al. / 2011(9)**	7	IVNCC-8 4V-4(50%) 3V-2(25%) LV-2(25%) With extra-ventricular NCC-7(4 patient had parenchymal and 3 had racemose NCC)	Mean=39 ±10	3(37.5%)-showed peripheral rim-like or nodular contrast enhancement	-	-
5.	**Nash TE et al. / 2018(30)**	23	LV-11(36.7%) 3V-2(6.7%) Aque-duct-1(3.3%) 4V-16(53.3%) With extraventricular NCC-17(73.9%)	Median = 31.8	-	-Hydrocephalus-17 (73.9%) -Ventriculi-tis-7(30.4%) -Entrapment of lateral ven-tricle-2(8.7%)	-Cyst excised -14(60.9%) -VP shunt -10(43.5%)
6.	**Present study**	10	LV-5(50%) 3V-1(10%) 4V-2(20%) 2 patients -had IVNCC in more than one ventricle With extra-ventricular NCC-0	37.2 ± 22.7	9-very thin smooth peripheral wall enhancement 1-thick irregular wall enhancement	Hydrocephalus –8(80%) Ependymitis -7(70%) Entrapment of lateral ven-tricle-1(10%)	Surgical treatment-6 Medical treatment -4

**Limitation: **Due to exclusion of patients with presence of intraventricular NCC along with the parenchymal form of NCC as well as IVNCC with racemose form of cysticercosis, the sample is limited to only isolated IVNCC in our study, and the sample size decreases. Still we tried to findout the visibility scores of T2W, 3D-DRIVE and SWI sequences for detection of wall and scolex of intraventricular NCC. Therefore, a larger study sample size is needed to confirm these added values of various MRI sequences in diagnosis, detection of cyst wall and scolex in isolated intraventricular NCC.

## Conclusion

Newer heavily T2-weighted MRI sequences (like 3D-DRIVE/FIESTA/CISS) and SWI improve the sensitivity of detection and localization of intraventricular neurocysticercosis, and further aid in guiding the management and neuroendoscopic cyst excision and further improvement of patients suffering from this disease. In a patient with an unexplained obstructive hydrocephalus especially from endemic regions of neurocysticercosis these newer MRI sequences may be added to conventional MRI sequences 
